# Disordered Microbial Communities in the Upper Respiratory Tract of Cigarette Smokers

**DOI:** 10.1371/journal.pone.0015216

**Published:** 2010-12-20

**Authors:** Emily S. Charlson, Jun Chen, Rebecca Custers-Allen, Kyle Bittinger, Hongzhe Li, Rohini Sinha, Jennifer Hwang, Frederic D. Bushman, Ronald G. Collman

**Affiliations:** 1 Department of Microbiology, University of Pennsylvania School of Medicine, Philadelphia, Pennsylvania, United States of America; 2 Department of Biostatistics and Epidemiology, University of Pennsylvania School of Medicine, Philadelphia, Pennsylvania, United States of America; 3 Pulmonary, Allergy and Critical Care Division, Department of Medicine, University of Pennsylvania School of Medicine, Philadelphia, Pennsylvania, United States of America; Charité, Campus Benjamin Franklin, Germany

## Abstract

Cigarette smokers have an increased risk of infectious diseases involving the respiratory tract. Some effects of smoking on specific respiratory tract bacteria have been described, but the consequences for global airway microbial community composition have not been determined. Here, we used culture-independent high-density sequencing to analyze the microbiota from the right and left nasopharynx and oropharynx of 29 smoking and 33 nonsmoking healthy asymptomatic adults to assess microbial composition and effects of cigarette smoking. Bacterial communities were profiled using 454 pyrosequencing of 16S sequence tags (803,391 total reads), aligned to 16S rRNA databases, and communities compared using the UniFrac distance metric. A Random Forest machine-learning algorithm was used to predict smoking status and identify taxa that best distinguished between smokers and nonsmokers. Community composition was primarily determined by airway site, with individuals exhibiting minimal side-of-body or temporal variation. Within airway habitats, microbiota from smokers were significantly more diverse than nonsmokers and clustered separately. The distributions of several genera were systematically altered by smoking in both the oro- and nasopharynx, and there was an enrichment of anaerobic lineages associated with periodontal disease in the oropharynx. These results indicate that distinct regions of the human upper respiratory tract contain characteristic microbial communities that exhibit disordered patterns in cigarette smokers, both in individual components and global structure, which may contribute to the prevalence of respiratory tract complications in this population.

## Introduction

Roughly one in five adults currently smoke cigarettes in the U.S.A. (www.cdc.gov/tobacco). Cigarette smoking is associated with an increased risk of acute respiratory tract infections [1], [2]. The upper airway serves as a site both for local upper respiratory tract infections, and for colonization by pathogenic microorganisms that can result in subsequent lower respiratory tract infection or invasive disease. Previous reports using limited culture-based methods have linked exposure to cigarette smoke with altered upper airway microbial colonization. Both active smoking in adults and passive exposure to cigarette smoke in children is associated with increased carriage of pathogenic organisms in the upper airways [3]. Cigarette smoke may promote pathogenic microbial colonization by enhancing bacterial binding to oral epithelial cells [4], disrupting effective nasal mucociliary clearance [5], [6], or impairing host immune responses against pathogens [7]. Cigarette smoke extract also differentially effects the survival of specific microbial species isolated from the human oral cavity, selecting for growth of gram negative bacteria such as *Pseudomonas aeruginosa* and *Klebsiella spp.* [8]. Cigarettes themselves harbor a broad range of potential pathogens, including *Acinetobacter*, *Bacillus*, *Burkholderia*, *Clostridium*, *Klebsiella*, *Pseudomonas aeruginosa*, and *Serratia* lineages [9] and may be a direct source of exposure to disease-causing organisms.

The ability of indigenous upper airway flora to interfere with pathogen colonization also plays an important role in microbial community homeostasis [10], [11], [12] and airway health. Past studies have shown that smoking can simultaneously deplete members of the normal commensal airway flora and enrich for potential pathogens. In the nasopharyx, smokers harbor fewer organisms with interfering capabilities and more disease-causing lineages than nonsmokers [13]. *Streptococcus pneumoniae*, *Haemophilus influenzae*, and *Moraxella catarrhalis* were more frequently isolated from nasopharyngeal swab cultures of smokers, while organisms that have been shown to limit the growth of these pathogens, including *Prevotella* and *Peptostreptococcus* species, were notably absent [13]. In the oral cavity, cigarette smoking enriches the subgingival microenvironment for organisms implicated in the pathogenesis of periodontitis [14], [15], including *Parvimonas*, *Fusobacerium*, *Bacteriodes*, *Prophyromonas*, and *Camplylobacter* species [14], [15]. After cessation of smoking, microbial communities are repopulated with a greater number of health-associated organisms and fewer potential pathogens in both the nasopharynx [16] and subgingiva [17]. Most of the above studies relied either on bacterial culture, which queries only a minority of the organisms present, or low throughput sequencing methods that identify only a modest subset of bacterial lineages, leaving the more global responses of bacterial communities to smoking only partially characterized.

Advances in deep sequencing and bioinformatics analyses now allow for comprehensive culture-independent analysis of human microbial communities. Sequencing and quantification of hypervariable regions of bacterial small subunit ribosomal RNA (16S rRNA) has enabled the unprecedented characterization of complex bacterial populations at diverse human body sites [18]. Recent studies have focused on the identification of the types, relative abundances, and variability of the healthy human microbiome to provide a foundation for comparison with disease. Such analyses implicate global alterations of microbial communities in the pathogenesis of asthma [19], cystic fibrosis [20], obesity [21], [22], and Crohn's disease [22].

To date, there have been no studies using deep sequencing technologies to assess the impact of cigarette smoking on airway microbial populations. Here we present the first intensive analysis of nasopharyngeal and oropharyngeal microbial communities from smokers and nonsmokers, employing multiplexed barcoded pyrosequencing of hypervariable regions of 16S rRNA. These results show a characteristic influence of smoking on global patterns of microbial communities, and identify bacterial taxa that best distinguish the oro- and nasopharyngeal microbial communities of cigarette smokers from nonsmokers.

## Results

### Study Population and Microbial Sequencing

Sixty-two adult participants were studied, including 29 current smokers and 33 nonsmokers. A subset of each group was sampled more than once (Table 1). All participants were free of clinical disease at the time of the sampling and none had used antibiotics within the past 3 months. The nonsmoker and smoker groups were similar in age but differed in gender (p<0.05). Sterile nylon-flocked swabs were used to sample the right and left nasopharynx and oropharynx of each participant separately.

**Table 1:** Characteristics of participants.

	Non Smokers	Smokers
**Total number of participants**	33	29
**Median age (range)**	28 years (22–51)	29 years (20–61)
**Sex (%male)**	58.9%	76.6%
**Pack years (mean+/−SD)**	n/a	11.82 years +/−13.13
**Median time from last cigarette (range)**	n/a	1.5hrs (1min–21hrs)
**Number of participants sampled more than once**	1	5

n/a, not applicable.

We isolated DNA from 291 swab samples. For each DNA sample, the variable region 1–2 (V1–V2) of the bacterial 16S rRNA gene was PCR-amplified using individually barcoded primer sets. We were unable to obtain amplification products from 1 nasopharyngeal sample. After multiplexed 454 pyrosequencing, we generated >813,700 high quality, partial (∼330bp) 16S rRNA gene sequences.

To avoid overestimation of bacterial diversity, pyrosequences were denoised prior to taxonomic assignment [23]. We identified >375,000 pre-cluster flowgrams, with an average of 1,335±603 (SD) per airway sample. Denoised sequences were analyzed using the Qiime pipeline [24], in which sequences were clustered at 97% sequence identity into operational taxonomic units (OTUs, also called phylotypes) and assigned a taxonomic identity by alignment to the RDP reference 16S rRNA database [25]. Using this analysis, we identified 1,720 and 1,973 OTUs in the right and left nasopharyngeal samples and 2,268 and 2,153 OTUs in the right and left oropharyngeal samples.

### Nasopharyngeal and Oropharyngeal Bacterial Diversity

The nasopharyngeal and oropharyngeal aggregate communities were characterized by a total of 381 different genera belonging to 11 different phlya.

The distribution of the top 4 phlya in the nasopharynx were *Firmicutes* (73%), *Proteobacteria* (12.6%), *Bacteroidetes* (7%), and *Actinobacteria* (5.6%); in the oropharynx the principal phyla were *Bacteroidetes* (36.4%), *Firmicutes* (27.7%), *Proteobacteria* (12.6%), and *Fusobacteria* (12.3%). *Streptococcus*, *Shigella*, *Acinetobacter*, and *Corynebacterium spp.* dominated nasopharyngeal communities, as well as environmentally linked *Leuconostoc*, *Lactococcus*, and *Weissella spp.* lineages found in dust and sterile swab samples. Oropharyngeal communities were dominated by *Prevotella*, *Fusobacterium*, *Neisseria*, *Leptotrichia*, and *Veillonella spp.*

We estimated the bacterial number and relative abundance within the oro- and nasopharynx by applying diversity estimators to our sampled communities. To account for heterogeneity in sequencing effort, all samples were analyzed by rarefaction and diversity measured at a common sampling depth (800 sequences). We then used the Chao 1 method to estimate the true population size for each airway site sampled and compared the number of different taxa found in the nasopharynx to the oropharynx, on both sides of the body. No consistent significant difference in the number of taxa between airway sites was found, indicating that there are no strong differences in bacterial richness between the nasopharyngeal and oropharyngeal microbial communities (*P* – value = 0.3142 left side, *P* – value = 0.0125 right side, two-sided Wilcoxon Rank Sum Test). We next used the Shannon Index to additionally account for taxa abundances in each community and compared estimates between airway sites. The Shannon index measures demonstrated greater bacterial diversity in the oropharynx when compared to the nasopharyngeal communities on both sides of the body (*P* – value = 4.72 E-11 left side, *P* – value = 8.67 E-14 right side, two-sided Wilcoxon Rank Sum Test). This analysis revealed that the oropharynx harbors a similar richness of lineages, but a more diverse microbiota than the nasopharynx.

**Figure 1:**
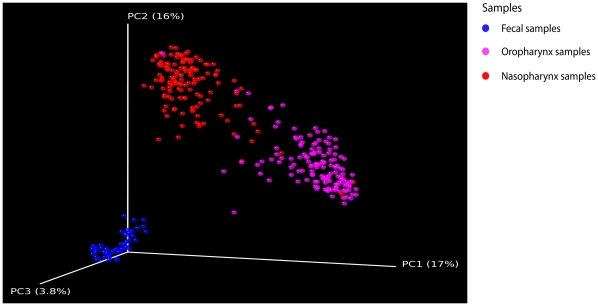
Comparison of bacterial community composition reveals that the upper airway microbiota is primarily structured by body habitat. Unweighted UniFrac was used to generated distances between oropharynx (red), nasopharynx (pink) and fecal (blue) microbiome samples, then scatterplots were generated using Principal Coordinate Analysis. The percentage of variation explained by each PCoA is indicated on the axes. The differences among communities from different body sites was significant with p<0.001 (t-test with permutation). Fecal microbial communities were from [27].

To explore potential relationships among airway communities, we quantified similarities between nasopharyngeal and oropharyngeal bacterial communities by calculating UniFrac distances [26]. Briefly, to compare two communities, 16S sequences for the two are aligned on a common phylogenetic tree, and the branch length unique to each community computed. A lower UniFrac value indicates that two communities contain phylogenetically more closely related organisms and thus are relatively more similar, whereas higher values indicate that more distantly related organisms populate the communities. Pairwise distances were calculated for all oropharyngeal and nasopharyngeal communities. For comparison, we also calculated pairwise distances for stool microbial communities obtained from an unrelated group of healthy human volunteers, from [27]. Visualization of clustering after principal coordinate analysis (PCoA) of the UniFrac distance matrix demonstrated strong clustering of communities by body site (Fig. 1). In contrast, the side of the body sampled had no apparent effect on bacterial community structure (P = 0.364 nasopharyngeal, P = 0.946 oropharyngeal, weighted UniFrac, PERMANOVA). Thus, the right and left samples provided a pair of replicates that could be compared in the subsequent analyses to assess reproducibility.

To identify lineages that distinguished between nasopharyngeal and oropharyngeal communities, we compared the abundance of each genus at all four airway sites using univariate tests of association (Wilcoxon Signed Rank or McNemar's test) (Table S2). After Bonferroni correction for multiple comparisons, a total of 81 bacterial taxa significantly varied between airway sites (Table S2). Many of these genera have been previously identified as normal residents of these airway habitats including *Propionibacterium*, *Corynebacterium*, and *Staphylococcus spp.*, in the nasopharynx [28] and *Neisseria*, *Haemophilus*, and anaerobic lineages such as *Prevotella*, *Veillonella*, and *Fusobacterium spp.* in the oropharynx [19], [29], [30].

### Effects of Cigarette Smoking on the Upper Airway Microbiome

**Figure 2:**
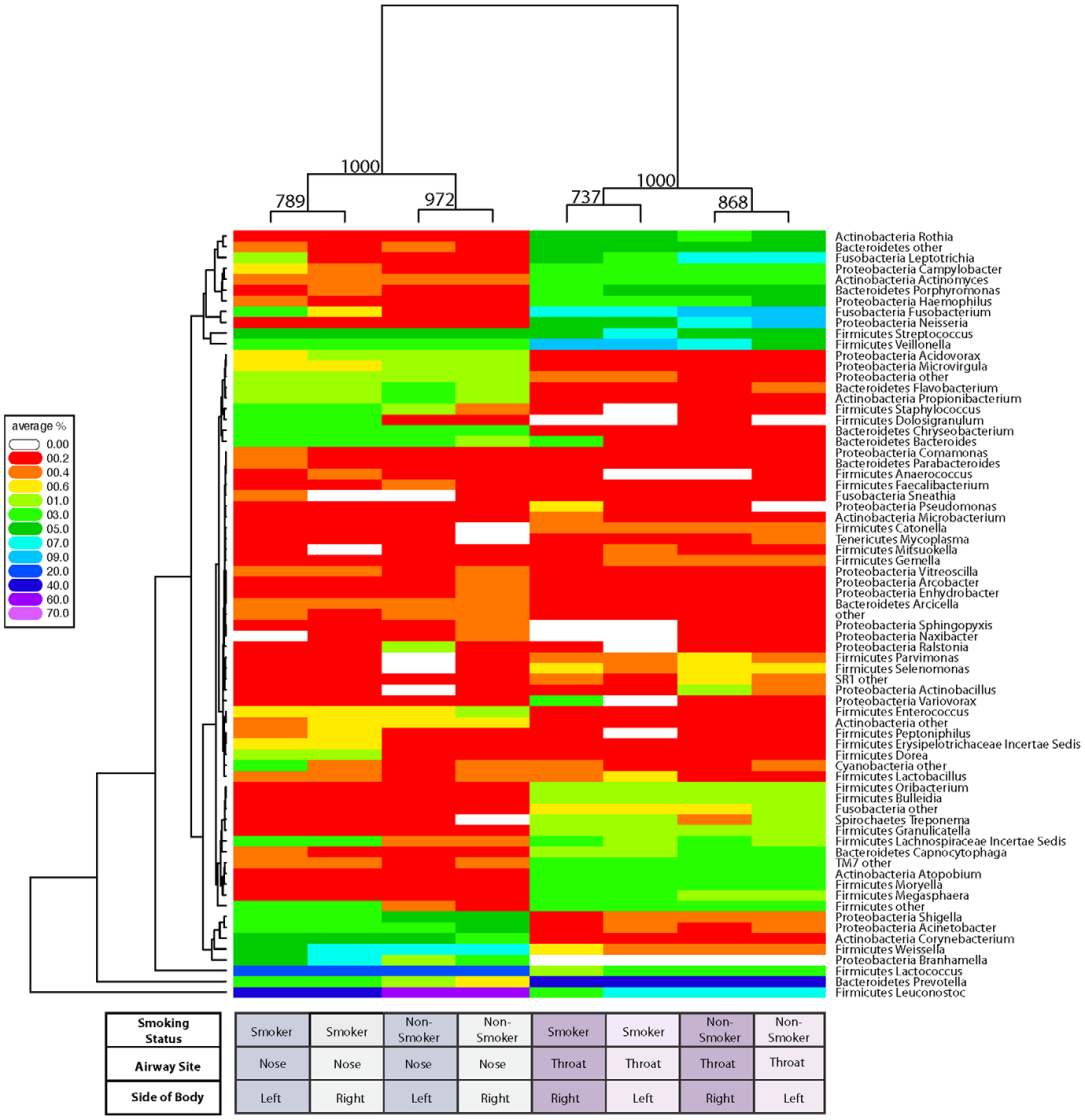
Analysis of abundances of bacterial lineages demonstrates that oro- and nasopharyngeal bacterial communities cluster based on smoking status. The relative abundance of each genus (rows) is shown by the key to the left of the figure. Communities are clustered by hierarchical clustering using complete linkage of Euclidean distance matrices. The number of times each split in the tree is seen in 1,000 bootstrapped samples is indicated at each node. The tree to the left of the heatmap groups genera together based on similarity of abundance profiles (i.e. if two genera are close in the tree, their abundance profiles across each airway site are similar).

To determine the relationship between airway bacterial communities and the impact of smoking, Euclidean distances were calculated based on sequence counts for each genus at all airway sites and used to perform hierarchical clustering. A total of 71 genera with an abundance of >0.2% in at least one airway site were included (Fig. 2). Bacterial communities clustered based on airway site (Fig. 2; bootstrap support 100%), as was the case with the UniFrac analysis (Fig. 1).

Within each airway habitat, bacterial communities from smokers clustered separately from nonsmokers, whereas communities from the right and left sides of the body demonstrated close similarity in genera abundances (Fig. 2; bootstrap support 78.9%–97.2% nasopharynx and 73.7–86.8% oropharynx). Thus the data sets for the two sides of the body independently replicate effects of smoking.

We next calculated the global differences between communities from smokers and nonsmokers using the unweighted (community membership) and weighted (community membership and relative abundance) UniFrac distance metrics (Table 2A and B). To determine the overall variance in the types and abundances of airway bacteria from smokers and nonsmokers, we compared the average UniFrac distance within smoking communities to the average distance within nonsmoking communities (within-group analysis). The different types of bacteria inhabiting both the oropharynx and nasopharynx varied more in smokers than nonsmokers (within-group unweighted UniFrac distance, P<0.05, permutation test) (Table 2A), indicating that microbial communities of smokers are overall more heterogeneous than those of nonsmokers.

**Table 2:** Distance-based ANOVA analysis: differences in bacterial community composition between smokers and nonsmokers.

A. Within-Group^1^	Nasopharynx		Oropharynx	
	*Right*	*Left*	*Right*	*Left*
**UnWeighted**	0.035	0.043	0.007	0.017
**Weighted**	0.037	0.202	0.6	0.811

B. Between vs Within-Group^2^	Nasopharynx		Oropharynx	
	*Right*	*Left*	*Right*	*Left*
**UnWeighted**	0.003	0.004	0.011	0.014
**Weighted**	0.066	0.172	0.0145	0.009

Table of *P*-values based on distance-based ANOVA with 10,000 label permutations comparing average UniFrac distances within **(A)** and between vs. within **(B)** bacterial microbiota from smoking and nonsmoking groups by airway site sampled. Significance threshold: *P*-value<0.05.

1 In all cases, bacterial communities from smokers had greater average within-group distances.

2 In all cases, bacterial communities from smokers had greater average between vs. within-group distances.

We then compared the average UniFrac distance within communities (either smokers or nonsmokers) to the average distance between pairs of communities where one was a smokers and the other a nonsmokers (within vs. between-group analysis). In the oropharynx, the microbiota of smokers and nonsmokers each formed separate clusters characterized by distinct types and abundances of bacterial lineages (P<0.05, unweighted and weighted UniFrac, distance-based ANOVA with permutation) (Table 2B). In the nasopharynx, communities of smokers were more similar in community membership to other smokers than to nonsmokers (P<0.05, unweighted UniFrac, PERMANOVA) (Table 2B).

### Taxa that Characterize the Upper Airway Microbiome of Cigarette Smokers

We next investigated the specific bacterial lineages that distinguished nonsmokers from smokers. The analysis was carried out separately for the left and right oropharynx and nasopharynx and results compared using univariate tests of association (Wilcoxon Rank Sum or Fisher's Exact t test). In the left oropharynx, 7 bacterial families were found to differ significantly between nonsmokers and smokers, of which 5 also differed on the right (P<0.05 Wilcoxon Rank Sum or Fisher's Exact t test) (Table 3A). Members of the *Megasphaera* and *Veillonella spp.* were most enriched for in both the right and left oropharynx of smokers, while *Capnocytophaga*, *Fusobacterium*, and *Neisseria spp.* significantly decreased in abundance (Table S3A). A greater number of families differed in the nasopharynx (12 on the right, and 16 on the left), with 8 families identified in both sides (Table 3B). Members of the *Eggerthella*, *Erysipelotrichaceae I.S.*, *Dorea*, *Anaerovorax*, and *Eubacterium spp*. were enriched, while *Shigella spp.* were decreased in both the right and left nasopharynx of smokers (Table S3B).

**Table 3:** Bacterial taxa that distinguish airway microbial communities of smokers from nonsmokers.

A. OROPHARYNX		NonSmokers vs. Smokers fold difference (*P-value*)	
Phyla	Family	Right	Left
**Actinobacteria**	Actinomycetaceae	–	1.18 (*0.039*)
**Bacteroidetes**	Porphyromonadaceae	–	0.81 (*0.0197*)
	Flavobacteriaceae	0.43 (*0.00155*)	0.48 (*0.00465*)
**Firmicutes**	Veillonellaceae	1.57 (*0.00108*)	1.89 (*0.000126*)
**Fusobacteria**	Fusobacteriaceae	0.69 (*0.00448*)	0.64 (*0.00185*)
**Proteobacteria**	Neisseriaceae	0.62 (*0.00116*)	0.58 (*0.00872*)
	Pasteurellaceae	0.51 (*0.0105*)	0.61 (*0.0498*)

B. NASOPHARYNX		NonSmokers vs. Smokers fold difference (*P-value*)	
Phyla	Family	Right	Left
**Actinobacteria**	Actinomycetaceae	0.91 (*0.0528*)	–
	Corynebacterineae	–	0.55 (*0.0375*)
	Coriobacteriaceae	–	0.812 (*0.0171*)
**Bacteroidetes**	Flexibacteraceae	0.67 (*0.0201*)	–
	Porphyromonadaceae	–	3.03 (*0.00846*)
	Flavobacteriaceae	–	0.86 (*0.0139*)
**Firmicutes**	Aerococcaceae	5.28 (*0.0363*)	18.00 (*0.00162*)
	Leuconostocaceae	0.79 (*0.0036*)	0.81 (0.0402)
	Eubacteriaceae	24.39 (*0.00428*)	13.78 (*0.000186*)
	Incertae Sedis XIII	25.61 (*0.00428*)	8.62 (*0.0165*)
	Lachnospiraceae	9.66 (*0.0000511*)	6.13 (*0.000101*)
	Peptostreptococcaceae	18.29 (*0.00965*)	–
	Ruminococcaceae	2.22 (*0.039*)	1.10 (*0.0297*)
	Erysipelotrichaceae	8.58 (*0.00218*)	3.54 (*0.0243*)
**Proteobacteria**	Rhodocyclaceae	0.32 (*0.0116*)	–
	Rhodobacteraceae	–	0.53 (*0.00756*)
	Enterobacteriaceae	0.54 (*0.0114*)	0.63 (*0.0291*)
	Alcaligenaceae	–	0.22 (*0.00332*)
	Methylophilaceae	–	0.19 (*0.0482*)
	Pasteurellaceae	–	9.81 (*0.0187*)

Bacterial families are grouped by phlya and listed in alphabetical order in the oropharynx **(A)** and nasopharynx **(B)**. Abundances and fold change of bacterial taxa were determined from pooled samples for the right and left oro- and nasopharynx. Family abundances were compared for each airway site from nonsmokers and smokers using univariate tests of association, either the Wilcoxon Rank Sum test or the Fisher's t test (for rare genera that can not be detected in at least half the samples from one location). Fold difference ratios >1 indicate a greater taxa abundance in smokers compared with nonsmokers (enriched for in smokers), fold difference ratios <1 indicate a decreased taxa abundance in smokers compared to nonsmokers (enriched for in nonsmokers). Only those families with *P*-values<0.05 are shown.

**Figure 3:**
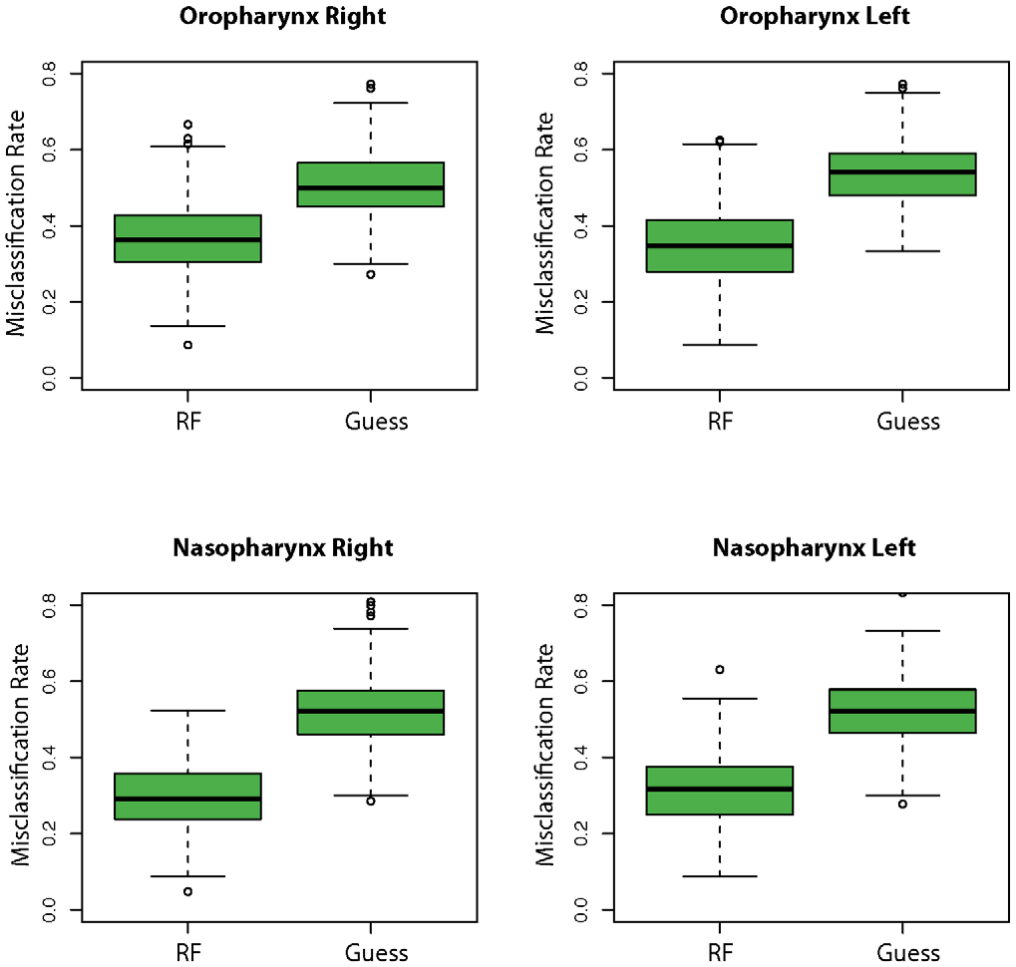
Partitioning airway microbial communities by smoking status using Random Forrest. Bacterial communities from each airway site were sorted by smoking status using the Random Forests trained algorithm and compared to guessing. Misclassification frequencies are plotted by airway site and side of body. RF = Random Forrest machine. Guess = guessing alone. The lower- and upper-most bars designate the lowest and highest value excluding outliers (defined as >1.5*IQR). The bottom and top of the green boxes denote the lower and upper hinge (close to 25% and 75% quantiles). The heavy black line designates the median misclassification frequency. The distribution of misclassification errors is significantly different between the two algorithms (*P* – value<2.2E-16 for all airway sites, Friedman Rank Sum test) and in all airway sites, Random Forests performs better than guessing (95% Confidence Interval: oropharynx right (−0.15–−0.13), oropharynx left (−0.20–−0.18); nasopharynx right (−0.23–−0.22), nasopharynx left (−0.22–−0.20).

We next identified those genera that best distinguish a smoker's bacterial community from that of a nonsmoker using a Random Forest supervised machine-learning algorithm. Abundances of all genera were determined for each sampled microbial community and used as input data sets for the algorithm. We fit a Random Forest model to training data sets consisting of bootstrapped samples of the original sample size, with the remaining unused samples used as a validation data set. The Random Forest consists of 500 classification trees with 20 genera evaluated at each node for all airway sites. Five hundred bootstrapped iterations are performed to obtain an estimate of the classification error rate. As shown in Fig. 3, the resulting models successfully partitioned microbial communities by smoking status with a median accuracy of 64% in the right and 65% in the left oropharynx, and 71% in the right and 68% in the left nasopharynx. For all four airway sites, we confirmed that the trained models were better able to assign microbial communities based on smoking status than by guessing alone (P<2.2E-16 at all airway sites, Friedman Rank Sum test, Fig. 3).

We then interrogated the specific organisms that differentiated smoker and nonsmoker microbiomes. The machine-learning algorithm revealed that in the oropharynx, *Capnocytophaga*, *Megasphaera*, *Veillonella*, *Haemophilus*, and *Neisseria spp.* best distinguished a smoker from a nonsmoker (ranked by mean Gini index value in Table S3A). In the nasopharynx, abundances of *Firmicutes* lineages including *Erysipelotrichaceace I.S.*, *Lachnospiraceae I.S.*, *Streptococcus*, and *Staphylococcus spp.* were most important for discriminating a smoker from a nonsmoker (Table S3B). Importantly, many of the genera identified by machine learning were also significantly associated with the upper respiratory tract populations of cigarette smokers by our univariate tests, and also demonstrated a high fold change in abundance compared with nonsmokers. These organisms included *Veillonella spp.* (increased in smokers) and *Fusobacterium spp.* (decreased) in the oropharynx, and *Erysipelotrichaceace I.S.* and *Lachnospiraceae I.S. spp.* (both increased) in the nasopharynx (Table S3A,B).

### Temporal Stability of Upper Airways Microbial Communities

Finally, we sampled the naso- and oropharynx of 6 people over multiple time points to characterize the stability of communities from the same person across time (over hours to weeks, for sampling time intervals see Table S4). For each airway site, we hypothesized that the average UniFrac distances between samples from the same subject taken over time would be significantly smaller (i.e. more similar) than the distances between samples from different subjects. Among the 6 people sampled more than once, we calculated the difference of average between vs. within subject distances for both the weighted and unweighted UniFrac values. In the oropharynx, microbial communities were more closely related within an individual at the latest time point than to those of other individuals (P-value<0.05, t-test with permutation, right and left sides). In the comparison to the oropharynx, nasopharyngeal community composition was less robust over time, but remained relatively stable when both lineage type and abundances are considered in the weighted analysis (P-value = 7 E-04 left, 0.255 right, t-test with permutation). Thus, upper respiratory tract microbial composition tends to be characteristic for each individual with little change over the time periods studied.

## Discussion

Here, we present the first comprehensive analysis of upper airway bacterial colonization in healthy adult cigarette smokers compared with nonsmokers using deep sequencing of microbial 16S rRNA genes. This study also sampled a relatively large number of participants (n = 62) compared to earlier studies [18], [31], [32]. In addition, by sampling the right and left sides of the body, we generated two independent data sets for each individual, which were found to be highly similar and thus provided important evidence for reproducibility and increased statistical power. Finally, our repeated sampling of a subset of participants allowed us to demonstrate that airway microbial communities within individuals were stable over the time period sampled (from hours to weeks), further supporting biological importance of the communities identified.

Although the nasopharynx and oropharynx are in open communication with each other and the environment, each sites harbored its own characteristic microbiota (Fig. 1 and Table S2). Similar to reports in other body sites, these two communities exhibit a stereotypical distribution of abundant taxa that are relatively conserved between people and within individuals over time [18]. The nasopharynx was characterized mainly by sequences related to members of the *Firmicutes* phyla (73%), with *Proteobacteria*, *Bacteriodetes*, and *Actinobacteria* members accounting almost all of the remaining sequences. This distribution of phyla is similar to, yet distinct from prior 16S rRNA sequencing studies of the anterior nares, which reported comparable groups [18], [31], [32] but a higher abundance of *Actinobacteria* more similar to skin microbiota [33], [34], perhaps because we sampled posterior nasopharyngeal organisms in addition to swab passage through the outer nares. In the oropharynx, bacterial sequences related to the *Bacteriodetes* phyla were most abundant at 36.4%, and members of the *Firmicutes* phyla were less represented at 27.7%, consistent with previous reports surveying the throat and oral cavity [18], [19], [29], [35]. *Proteobacteria* taxa were represented in similar proportions in both airway sites (∼12%). In this study, we detected a somewhat higher percentage of members of the *Fusobacteria* phyla at 12.3% compared to other 16S rRNA pyrosequencing surveys [29]. In addition, we were able to identify several unusual lineages, such as *Bergeyella spp.*, that have been linked to human disease in clinical case reports [36] and only recently have been associated with the airways microbiota by culture-independent surveys [20], [37]. We were also able to detect a considerable abundance of apparent anaerobes, such as *Prevotella*, *Capnocytophaga*, and *Rothia spp.*, as well as lesser characterized *Atopobium*, *Peptoniphilus*, and *Selenomonas spp.* amongst others. As culture-dependent studies are mostly restricted to the detection of aerobic bacteria, the contribution of anaerobic lineages to community composition and dynamics has been largely unstudied.

Our main findings center on the identification of microbial community patterns and specific bacterial groups that are altered by smoking. Attention has previously focused on how smoking affects carriage of known bacterial pathogens, as well as the presence of specific commensal organisms. Those studies that have addressed alterations to normal flora have investigated a select group of organisms shown to have an impact on host resistance to pathogen colonization [10], [11], [12] and demonstrated that smokers have altered carriage of these commensal lineages [13], [15]. However, beyond a small cohort of potential interfering organisms, the presence of a robust endogenous microbial community may also regulate pathogenic colonization, but this has not been addressed in a global manner. We found that smokers' upper respiratory tract communities were significantly more diverse than those of non-smokers, suggesting degradation of normal community structure. It will be important in future studies to determine whether such a disruption of normal colonization patterns in smokers contributes to infectious complications and/or more efficient pathogen colonization.

In investigating specific lineages that distinguished smokers upper respiratory tract from nonsmokers, we used both a univariate analysis and a machine learning approach. We found a greater abundance of both known pathogens, and organisms not previously recognized as associated with disease in smokers. In the oropharynx, the greatest increase in smokers compared with nonsmokers was in *Megasphaera spp.*, an anaerobic gram negative lineage of the *Firmicutes* phyla which are known to reside in the oral cavity and is associated with periodontitis [38]. Overall, 15 bacterial genera containing potential pathogens increased in abundance in either the univariate statistical analysis or were identified as discriminators in the machine learning study, including *Streptococcus*, *Veillonella*, *Actinomyces* and *Atopobium spp.* (Table S3A). In contrast, the *Peptostreptococcus* genera was decreased in smokers, which may be significant because several species are implicated as an interfering bacteria, known to inhibit growth of pathogenic bacteria in the upper respiratory tract [39]. Several additional members of the normal oral microbiota were also decreased in abundance, including *Capnocytophaga*, *Fusobacterium*, and *Neisseria spp.*

In the nasopharynx, previous literature implicated *Haemophilus influenzae* non-type B as increased in smokers [13], and we saw an increase in *Haemophilus spp.* in smokers (Table S3B) (although on one body side only, suggesting a relatively modest association). Our data also identified several genera with substantial increased abundance in smokers that have not been noted previously. In the nasopharynx, these included *Eggerthella*, *Erysipelotrichaceae I.S.*, *Dorea*, *Anaerovorax*, and *Eubacterium spp*. All of these genera contain gram-positive anaerobic lineages, and clinical isolates of *Eubacterium spp.* have been previously associated with active oral infections [40]. In addition, we demonstrated a large increase in *Abiotrophia spp.*, which can be isolated from dental plaque [41], [42] and is an occasional cause of bacterial endocarditis [42]. Interestingly, only *Shigella spp.* were decreased in nasopharyngeal communities of smokers compared with nonsmokers. Together, these data suggest that smoking increased the burden of gram-positive anaerobic bacteria in the nasopharynx, some of which have been associated with disease.

To date, the effects of cigarette smoke on altering microbial colonization have been characterized in greatest detail in the subgingival environment, especially as it relates to periodontitis [14], [15], [17], [43]. A recent study using 16S rRNA terminal restriction fragment length polymorphism analysis of subgingival communities, found significantly different microbial profiles between smokers and nonsmokers [17]. Subsequent reports using 16S rRNA sequence profiling of subgingival plaque identified an increase in several disease-associated organisms in smokers, including *Parvimonas*, *Fusobacterium*, *Campylobacter*, *Bacteroides*, *Dialister*, and *Treponema spp.* and a decrease in potential health-promoting taxa from the *Veillonella*, *Neisseria*, *Streptococcus*, and *Capnocytophaga* genera [43]. Here, we detected comparable effects of smoking on airway flora, such as a decrease in *Neisseria* and *Capnocytophaga spp.* in the oropharynx and an increase in *Campylobacter spp.* in the nasopharynx (Table S3A, 3B). Also similar to subgingival environments, members of the *Bacteroides* and *Dialister* genera were identified by machine learning as particularly important for distinguishing the microbiota of a smoker in the oropharynx (Table S2A). In contrast to those reports on oral communities, we detected a decrease in *Fusobacterium spp.* and an increase in *Streptococcus* and *Veillonella spp.* in the oropharynx of smokers (Table S3A), which is likely attributable to differences in the subgingival versus naso-/oropharyngeal microbial environments.

Thus, our findings identify characteristic patterns of upper respiratory microbial communities in smoking and nonsmoking healthy adults, and define a collection of changes in smokers that suggests both aberrant global community structure and differences in specific organisms. These alterations in healthy smokers may reflect pathogenic processes contributing to the enhanced risk of upper and lower respiratory tract infection associated with cigarette smoking.

## Materials and Methods

### Ethics Statement

The Institutional Review Board of the University of Pennsylvania approved all study protocols and all participants provided written, informed consent (protocol #810987).

### Subjects and Sample Collection

Healthy adults were recruited to provide samples over a four-month period from December 2009–March 2010 from Philadelphia, PA. Smokers were defined as current smoking of >2 cigarettes daily for more than 6 months, and nonsmokers were defined as less than 100 cigarettes lifetime. Individuals with known chronic health conditions or with respiratory tract symptoms within 12 weeks prior to study were excluded, and none of the subjects had used antibiotics within the past 3 months. The health and smoking status of the volunteers was self-reported. Participants were asked to avoid eating or drinking for one hour prior to sampling. The nasopharynx and oropharynx were sampled using nylon-flocked swabs (Copan). The right and left posterior oropharynx were sampled trans-orally adjacent to the tonsillar pillars, and the right and left nasopharynx were sampled through the nares. After collection, swabs were immediately cut into MoBio 0.7 mm garnet bead tube (Mo Bio Laboratories) using autoclaved and flamed scissors in a biosafety cabinet, placed at −80°C within 1 hour, and stored for <1 week prior to DNA extraction. See Table S1 for a summary of samples used in this study.

### DNA Extraction and Purification

Genomic DNA was extracted from swabs using the QIAamp DNA Stool Minikit (Qiagen) with the following modifications. 1500ul of ASL buffer and 5mM DTT was added to the nylon tips of frozen swabs that had been cut into beadbeater tubes. Tubes were beadbeat using BioSpec Products Inc. Minibeadbeater-16 for 1 min and incubated at 95°C for 10 min. The remaining steps were preformed as per manufacturer protocol. DNA was eluted with 100 uL buffer EB (Qiagen) and stored at −20°C.

### PCR amplification of the V1V2 Region of Bacterial 16S rRNA Genes

For each sample, we amplified the 16s rRNA gene using the reverse primer 5′-GCCTCCCTCGCGCCATCAGNNNNNNNN *CTGCTGCCTYCCGTA*-3′ and the forward primer 5′-GCCTTGCCAGCCCGCTCAG *AGAGTTTGATCCTGGCTCAG*-3′ . The underlined sequences are the 454 Life Sciences primer B (forward) and A (reverse). The italicized sequence is the broad range bacterial primer BSR357 (reverse) and BSF8 (forward). Each reverse primer contained a unique 8-nt error-correcting Hamming barcode (designated by NNNNNNNN) used to tag each PCR product. Duplicate 25uL reactions were carried out with AccuPrime Taq DNA Polymerase High Fidelity (Invitrogen) under the following reaction conditions: 2.5 uL 10× Buffer 2, 0.4 uL Taq, 11.1 uL PCR-grade H_2_O, 0.5 uL forward primer and 0.5 uL reverse primer (20 pmol/uL each) and 10 uL template DNA. PCR reactions were assembled in a PCR bay in which all surfaces and pipettes had been decontaminated with DNA AWAY (Molecular BioProducts). Reactions were run on a Applied Biosystems Veriti thermocycler with the following cycling conditions: initial denaturing at 95°C for 5 min followed by 30 cycles of denaturation at 95°C for 30 seconds, annealing at 56°C for 30 seconds, and extension at 72 C for 90 seconds, with a final extension of 8 min at 72°C. Replicate amplicons were pooled and visualized on 0.8% agarose gels containing ethidium bromide. Amplicons were bead purified using Agencourt AMPure XP (Beckman Coulter) as per manufacturer instructions.

### 454 Pyrosequencing and Sequence Analysis

Purified amplicons were quantified using Quant-iT PicoGreen kit (Invitrogen) and pooled in equimolar ratios. Pyrosequencing was carried out using primer A and the Titanium amplicon kit on a 454 Life Sciences Genome Sequencer FLX instrument (Roche). Pyrosequence reads were denoised with the denoising algorithim described by *Quince et al* [23], [44], including removing sequences with a mean window quality score <25. Barcoded 16S rRNA sequences were then uploaded into QIIME and processed as described by *Caporaso et al.* [24]. QIIME removes sequences from the analysis if they were <200 or >800 nt, had a quality score <25, uncorrectable barcodes, contained ambiguous bases or mismatches in the primer sequences, and if they had a homopolymer run >6 nt. Sequence reads were then clustered into OTUs at 97% sequence identity with UCLUST [45], aligned to full length 16S rRNA sequences with PyNAST [46], assigned a taxonomic identity with the Ribosomal Database Project classifier (minimum support threshold of 50%) [25], and used to construct phylogenetic trees using FastTree2 [47]. QIIME generates data summaries of the proportions of identified taxa in each community and calculates the amount of bacterial diversity shared between two communities using the UniFrac metric [26]. Clustering was visualized for the weighted UniFrac analysis using Principal Coordinates Analysis.

As controls, 5 sterile swabs and 2 swabs of autoclaved and flamed scissors were also tested, handled under identical conditions. The sterile swab and scissor samples yielded three predominant lineages (>15% abundance), which were assigned by RDP to the genera *Lactococcus*, *Weissella*, and *Leuconostoc* of the *Firmicutes* phyla. *Lactococcus spp.* have been associated with indoor dust in previous literature [48], [49], [50]. *Weissella spp.* and *Leuconostoc spp.* have also been associated with environmental habitats [51]. These lineages were also abundant in nasopharyngeal samples, particularly *Lactococcus* and *Leuconostoc* (>15% abundance).

### Statistical methods

Clinical characteristics were compared as mean, standard deviation, median, range, counts and percentages. Significant changes in lineage abundance between groups were assessed using univariate statistical tests: Wilcoxon Rank Sum test and Wilcoxon Signed rank test or Fisher's exact test and McNemar's test if the taxon cannot be detected in more than half of the samples from one location. Clustering of groups was performed on the Euclidean distance matrix using hierarchical clustering with complete linkage (“hclust” function in R). Confidence of the clustering pattern was assessed by bootstrapping the samples in each group 1,000 times. UniFrac [26], [52] was used to measure beta diversity between all pairs of bacterial communities, including both an unweighted (considers only presence or absence of lineages to assess community membership) and a weighted analysis (includes relative abundances of lineages to assess community structure). To test for differences in community composition between various sample groups, we used Permutational Multivariate Analysis of Variance based on the UniFrac distance matrix (PERMANOVA ,“adonis” function in the “vegan” package of R). To test for the difference of within-group distance for two groups, we used the difference of within-group distance means as the test statistic. Statistical significance was assessed using 10,000 permutations of sample labels. A learning machine was trained using the Random Forest algorithm with prediction accuracy assessed using an out-of-bag estimation (“randomForest” package in R). The distribution of misclassification errors between the trained machine and simple guess (the class label was predicted based on the majority class in the training data set) were compared by the Friedman Rank Sum test.

## Supporting Information

Table S1 **Summary of samples used in the study.** DNA was amplified using the BSF8/BSR357 16S primer pair, purified using magnetic beads, and sequenced in the reverse direction using the FLX platform.
(XLS)

Table S2 **Bacterial taxa that vary by airway site.** Bacterial genera are grouped by phyla. Abundances and fold differences of bacterial taxa were determined from pooled samples for the right and left oro- and nasopharynx and then averaged over the side of the body sampled for both airway sites. Genera abundances were compared for significant changes from the oropharynx to the nasopharynx using univariate tests of association, either the Signed Rank test or the McNemar test (for rare genera that cannot be detected in at least half the samples from one location). Fold difference ratios >1 indicate a greater taxa abundance in the nasopharynx compared to oropharynx (enriched for in the nasopharynx), fold difference ratios <1 indicate a decreased taxa abundance in nasopharynx compared to oropharynx (enriched for in the oropharynx). Only those taxa with >10-fold change in abundance are listed.
(XLS)

Table S3 **Bacterial genera that distinguish the airway microbial communities of a nonsmoker from a smoker.** Bacterial genera are grouped by phlya and listed in alphabetical order in the oropharynx **(A)** and nasopharynx **(B)**. Abundances and fold change of bacterial taxa were determined from pooled samples for the right and left oro- and nasopharynx. Genera abundances were compared for significant changes from each airway site from nonsmokers to smokers using univariate tests of association, either the Wilcoxon Rank Sum test or the Fisher's t test (for rare genera that can not be detected in at least half the samples from one location). Fold difference ratios >1 indicate a greater taxa abundance in smokers compared to nonsmokers (enriched for in smokers), fold difference ratios <1 indicate a decreased taxa abundance in smokers compared to nonsmokers (enriched for in nonsmokers). Only those genera with *P*-values<0.05 are shown. Bacterial taxa important for distinguishing a microbial community of a smoker from a nonsmoker by Random Forest machine learning are ranked by their mean Gini index value (the relative weight of each taxa to the classification prediction). Taxa that best distinguish a smoking from a nonsmoking bacterial community have a higher index value.
(XLS)

Table S4 **Summary of samples taken from the same person over time.** Sample index 1 was taken at time point 0. All subsequent samples from the same patient are denoted by increasing sample index number with time in hours from the first sample.
(XLS)

## References

[1] Aronson MD, Weiss ST, Ben RL, Komaroff AL (1982). Association between cigarette smoking and acute respiratory tract illness in young adults.. JAMA.

[2] Turkeltaub PC, Gergen PJ (1991). Prevalence of upper and lower respiratory conditions in the US population by social and environmental factors: data from the second National Health and Nutrition Examination Survey, 1976 to 1980 (NHANES II).. Ann Allergy.

[3] Brook I, Gober AE (2008). Recovery of potential pathogens in the nasopharynx of healthy and otitis media-prone children and their smoking and nonsmoking parents.. Ann Otol Rhinol Laryngol.

[4] El Ahmer OR, Essery SD, Saadi AT, Raza MW, Ogilvie MM (1999). The effect of cigarette smoke on adherence of respiratory pathogens to buccal epithelial cells.. FEMS Immunol Med Microbiol.

[5] Stanley PJ, Wilson R, Greenstone MA, MacWilliam L, Cole PJ (1986). Effect of cigarette smoking on nasal mucociliary clearance and ciliary beat frequency.. Thorax.

[6] Tamashiro E, Xiong G, Anselmo-Lima WT, Kreindler JL, Palmer JN (2009). Cigarette smoke exposure impairs respiratory epithelial ciliogenesis.. Am J Rhinol Allergy.

[7] Arcavi L, Benowitz NL (2004). Cigarette smoking and infection.. Arch Intern Med.

[8] Ertel A, Eng R, Smith SM (1991). The differential effect of cigarette smoke on the growth of bacteria found in humans.. Chest.

[9] Sapkota AR, Berger S, Vogel TM (2010). Human pathogens abundant in the bacterial metagenome of cigarettes.. Environ Health Perspect.

[10] Crowe CC, Sanders WE, Longley S (1973). Bacterial interference. II. Role of the normal throat flora in prevention of colonization by group A Streptococcus.. J Infect Dis.

[11] Brook I, Gober AE (1995). Role of bacterial interference and beta-lactamase-producing bacteria in the failure of penicillin to eradicate group A streptococcal pharyngotonsillitis.. Arch Otolaryngol Head Neck Surg.

[12] Aly R, Maibach HI, Shinefield HR, Mandel A, Strauss WG (1974). Bacterial interference among strains of Staphylococcus aureus in man.. J Infect Dis.

[13] Brook I, Gober AE (2005). Recovery of potential pathogens and interfering bacteria in the nasopharynx of smokers and nonsmokers.. Chest.

[14] van Winkelhoff AJ, Bosch-Tijhof CJ, Winkel EG, van der Reijden WA (2001). Smoking affects the subgingival microflora in periodontitis.. J Periodontol.

[15] Zambon JJ, Grossi SG, Machtei EE, Ho AW, Dunford R (1996). Cigarette smoking increases the risk for subgingival infection with periodontal pathogens.. J Periodontol.

[16] Brook I, Gober AE (2007). Effect of smoking cessation on the microbial flora.. Arch Otolaryngol Head Neck Surg.

[17] Fullmer SC, Preshaw PM, Heasman PA, Kumar PS (2009). Smoking cessation alters subgingival microbial recolonization.. J Dent Res.

[18] Costello EK, Lauber CL, Hamady M, Fierer N, Gordon JI (2009). Bacterial community variation in human body habitats across space and time.. Science.

[19] Hilty M, Burke C, Pedro H, Cardenas P, Bush A (2010). Disordered microbial communities in asthmatic airways.. PLoS One.

[20] Cox MJ, Allgaier M, Taylor B, Baek MS, Huang YJ (2010). Airway microbiota and pathogen abundance in age-stratified cystic fibrosis patients.. PLoS One.

[21] Turnbaugh PJ, Hamady M, Yatsunenko T, Cantarel BL, Duncan A (2009). A core gut microbiome in obese and lean twins.. Nature.

[22] Dicksved J, Halfvarson J, Rosenquist M, Jarnerot G, Tysk C (2008). Molecular analysis of the gut microbiota of identical twins with Crohn's disease.. ISME J.

[23] Quince C, Lanzen A, Curtis TP, Davenport RJ, Hall N (2009). Accurate determination of microbial diversity from 454 pyrosequencing data.. Nat Methods.

[24] Caporaso JG, Kuczynski J, Stombaugh J, Bittinger K, Bushman FD (2010). QIIME allows analysis of high-throughput community sequencing data.. Nat Methods.

[25] Wang Q, Garrity GM, Tiedje JM, Cole JR (2007). Naive Bayesian classifier for rapid assignment of rRNA sequences into the new bacterial taxonomy.. Appl Environ Microbiol.

[26] Lozupone C, Knight R (2005). UniFrac: a new phylogenetic method for comparing microbial communities.. Appl Environ Microbiol.

[27] Wu GD, Lewis JD, Hoffmann C, Chen YY, Knight R (2010). Sampling and pyrosequencing methods for characterizing bacterial communities in the human gut using 16S sequence tags.. BMC Microbiol.

[28] Rasmussen TT, Kirkeby LP, Poulsen K, Reinholdt J, Kilian M (2000). Resident aerobic microbiota of the adult human nasal cavity.. APMIS.

[29] Jakobsson HE, Jernberg C, Andersson AF, Sjolund-Karlsson M, Jansson JK (2010). Short-term antibiotic treatment has differing long-term impacts on the human throat and gut microbiome.. PLoS One.

[30] Klepac-Ceraj V, Lemon KP, Martin TR, Allgaier M, Kembel SW (2010). Relationship between cystic fibrosis respiratory tract bacterial communities and age, genotype, antibiotics and Pseudomonas aeruginosa.. Environ Microbiol.

[31] Wos-Oxley ML, Plumeier I, von Eiff C, Taudien S, Platzer M (2010). A poke into the diversity and associations within human anterior nare microbial communities.. ISME J.

[32] Lemon KP, Klepac-Ceraj V, Schiffer HK, Brodie EL, Lynch SV (2010). Comparative analyses of the bacterial microbiota of the human nostril and oropharynx.. MBio.

[33] Grice EA, Kong HH, Conlan S, Deming CB, Davis J (2009). Topographical and temporal diversity of the human skin microbiome.. Science.

[34] Grice EA, Kong HH, Renaud G, Young AC, Bouffard GG (2008). A diversity profile of the human skin microbiota.. Genome Res.

[35] Zaura E, Keijser BJ, Huse SM, Crielaard W (2009). Defining the healthy “core microbiome” of oral microbial communities.. BMC Microbiol.

[36] Han YW, Shen T, Chung P, Buhimschi IA, Buhimschi CS (2009). Uncultivated bacteria as etiologic agents of intra-amniotic inflammation leading to preterm birth.. J Clin Microbiol.

[37] Armougom F, Bittar F, Stremler N, Rolain JM, Robert C (2009). Microbial diversity in the sputum of a cystic fibrosis patient studied with 16S rDNA pyrosequencing.. Eur J Clin Microbiol Infect Dis.

[38] Kumar PS, Griffen AL, Moeschberger ML, Leys EJ (2005). Identification of candidate periodontal pathogens and beneficial species by quantitative 16S clonal analysis.. J Clin Microbiol.

[39] Brook I (2005). The role of bacterial interference in otitis, sinusitis and tonsillitis.. Otolaryngol Head Neck Surg.

[40] Downes J, Munson MA, Spratt DA, Kononen E, Tarkka E (2001). Characterisation of Eubacterium-like strains isolated from oral infections.. J Med Microbiol.

[41] Mikkelsen L, Theilade E, Poulsen K (2000). Abiotrophia species in early dental plaque.. Oral Microbiol Immunol.

[42] Senn L, Entenza JM, Greub G, Jaton K, Wenger A (2006). Bloodstream and endovascular infections due to Abiotrophia defectiva and Granulicatella species.. BMC Infect Dis.

[43] Shchipkova AY, Nagaraja HN, Kumar PS (2010). Subgingival Microbial Profiles of Smokers with Periodontitis.. J Dent Res.

[44] Reeder J, Knight R (2010). Rapidly denoising pyrosequencing amplicon reads by exploiting rank-abundance distributions.. Nat Methods.

[45] Edgar RC (2010). Search and clustering orders of magnitude faster than BLAST.. Bioinformatics.

[46] Caporaso JG, Bittinger K, Bushman FD, DeSantis TZ, Andersen GL (2010). PyNAST: a flexible tool for aligning sequences to a template alignment.. Bioinformatics.

[47] Price MN, Dehal PS, Arkin AP (2010). FastTree 2–approximately maximum-likelihood trees for large alignments.. PLoS One.

[48] Kaarakainen P, Rintala H, Vepsalainen A, Hyvarinen A, Nevalainen A (2009). Microbial content of house dust samples determined with qPCR.. Sci Total Environ.

[49] Pakarinen J, Hyvarinen A, Salkinoja-Salonen M, Laitinen S, Nevalainen A (2008). Predominance of Gram-positive bacteria in house dust in the low-allergy risk Russian Karelia.. Environ Microbiol.

[50] Rintala H, Pitkaranta M, Toivola M, Paulin L, Nevalainen A (2008). Diversity and seasonal dynamics of bacterial community in indoor environment.. BMC Microbiol.

[51] Yang J, Cao Y, Cai Y, Terada F (2010). Natural populations of lactic acid bacteria isolated from vegetable residues and silage fermentation.. J Dairy Sci.

[52] Lozupone CA, Hamady M, Kelley ST, Knight R (2007). Quantitative and qualitative beta diversity measures lead to different insights into factors that structure microbial communities.. Appl Environ Microbiol.

